# Influence of timing of sexual debut and first marriage on sexual behaviour in later life: findings from four survey rounds in the Kisesa cohort in northern Tanzania

**DOI:** 10.1136/sti.2008.033704

**Published:** 2009-03-13

**Authors:** B Żaba, R Isingo, A Wringe, M Marston, E Slaymaker, M Urassa

**Affiliations:** 1 TAZAMA Project, National Institute for Medical Research, Mwanza, Tanzania; 2 Centre for Population Studies, London School of Hygiene and Tropical Medicine, London, UK

## Abstract

**Objectives:**

To evaluate quality of sexual debut and first marriage data, measure trends and study the association of risky sexual behaviour in youth with adult risk behaviour.

**Methods:**

Reports on age at first sex (AFS) and age at first marriage (AFM) from the Kisesa cohort study, 1994–2004, were evaluated for consistency and used to describe trends in median age-at-event and time spent single but sexually active in different birth cohorts. The association of these variables with marital stability and numbers of partners at later ages was explored using statistical regression techniques.

**Results:**

AFS and AFM were inconsistently reported by 32% and 33% of respondents, respectively, but there was no general tendency to report lower or higher ages at a later report date. In 10-year birth cohorts born between 1950–9 and 1980–9, male median AFS declined from 18.1 to 17.0 years and female median AFM rose from 16.2 to 16.6 years. Young people of both sexes currently spend longer sexually active but unmarried than previously. Early marriage is statistically associated with remarriage and polygamy; longer time between sexual debut and marriage is associated with higher numbers of partners at later stages of life.

**Conclusion:**

Inconsistent reporting of age-at-event introduces noise but does not bias estimates of population level indicators. Lengthening time spent single and sexually active suggests that men and women entering first marriage will have been exposed to increased numbers of non-marital partners. Successful youth interventions may also influence adult behaviour.

Sexual abstinence before marriage and being faithful within marriage are HIV prevention messages that have been widely promoted across Africa,^1^ ^2^ with the result that many prevention campaigns have a strong focus on youth. Uganda and Zimbabwe have recorded increases in age at sexual debut that coincided with declines in HIV prevalence,^3^ ^4^ fuelling an ongoing debate as to the importance of abstinence in HIV prevention^5^^–^7 and the reliability of data on sexual behaviour.^8^^–^15 Tanzania has also experienced a modest decline in HIV prevalence, from over 8% in 1995 to around 6.5% in 2005,^16^ ^17^ but this has not been accompanied by an increase in age at sexual debut.^8^ ^18^

Family planning campaigns have also advocated a later start to sexual activity and marriage in order to avoid teenage pregnancy and its unfavourable health and social consequences.^19^ ^20^ Age at first marriage has been investigated in relation to HIV infection, with some researchers suggesting that early marriage increases the risk of HIV, especially for women,^21^ and others arguing that late marriage—usually the result of a long gap between sexual debut and first marriage—increases risk.^22^

The time spent between sexual debut and marriage is considered a high-risk period because it is a time for sexual experimentation, and the partnerships formed by young people during this time are often transitory.^23^^–^26 Sexually transmitted infections acquired by an individual during this time could pose a health risk to the future spouse, even if both partners were faithful during the subsequent marriage.^27^^–^33 Furthermore, it has been postulated that sexual behaviour patterns established at this time may influence behaviour in later life.^34^^–^37

The aim of this paper is threefold: (1) to examine the quality of reporting of age at first sex (AFS) and age at first marriage (AFM) in a long standing cohort study in Tanzania by comparing reports for individuals who participated in two or more survey rounds; (2) to describe trends over time in sexual debut and first marriage by comparing median ages at these transition events for birth cohorts of men and women who participated in the study; and (3) to examine the relationship between these aspects of premarital sexual behaviour and behaviour in later life.

## METHODS

### Field work

The Kisesa open cohort study has been described in earlier publications.^38^^–^41 Briefly, it covers six villages and a roadside trading centre which make up Kisesa ward in Magu district. Kisesa is part of Mwanza region in north-west Tanzania, located about 20 km east of Mwanza City, the second largest city in Tanzania, lying on the main road to the Kenyan border. Demographic surveillance has been conducted at approximately half-yearly intervals since 1994 by means of short household visits that simply update the population rosters. HIV status and sexual behaviour data are collected at more detailed surveys, approximately 3 years apart, in village-based survey centres to which all adults aged ⩾15 years are invited.^42^ This analysis considers data collected in the first four of these surveys conducted in 1994, 1997, 2000 and 2003.

Questions on sexual activity status (“Have you ever had sex?”) and marital status were asked at each of the surveys. The first and last surveys additionally collected information about AFS and AFM for qualifying individuals. Quality of reporting was assessed at each round by looking for illogical transitions (eg, from married at an earlier round to never married at a later round) and reported ages at events were compared for rounds 1 and 4 for those who participated in both rounds and had already experienced the event at round 1. Age-at-event reporting at round 4 was also checked against current status reports at earlier rounds.

A 1-year discrepancy in reported event ages and age at survey was allowed because many individuals do not know their exact date of birth (only their year of birth), so that estimated ages at surveys based on interview date may be out by 1 year depending on the survey date and actual (unknown) birth month. Age reports differing by 1 year are thus classified as consistent and those differing by ⩾2 years are classified as inconsistent.

### Descriptive analyses

Life table methods were used to estimate median AFS and median AFM.^8^ Current status data (never had sex, never married) were used to provide information on exposure to risk and censoring, even for those who had never provided direct information on event ages. Conflicting age reports from rounds 1 and 4 were averaged to obtain corrected age-at-event for those with inconsistent reports. Estimated event ages were calculated for those few individuals who did not directly report an age-at-event but who were recorded as experiencing a transition in current status (eg, from never married to currently married) between rounds 1 and 2, or rounds 2 and 3, by averaging reporting ages either side of the transition interval. Median AFS and AFM were computed for men and women based on 10-year birth cohorts, and trends were compared for all reports (including estimates) and consistent reports only.

The reported number of partners in the last 12 months could not be subjected to the same kind of consistency checks, but trends in this aspect of behaviour were compared across surveys for all respondents classified by sex, birth cohort and marital status (never/ever married). We calculated years between sexual debut and first marriage (or sexual debut and current age for never married respondents), which represents the length of premarital sexual activity. For the age group 15–24 years in which the majority of events occur, we compared person-years spent as a virgin, sexually active but unmarried and ever married for different birth cohorts.

### Regression analyses

To investigate whether the timing of transitions at the start of sexually active life influences later risk exposure, we used logistic regression analysis in which we examined the odds of experiencing the break-up of a first marriage using AFS and time spent between sexual debut and first marriage as predictor variables. Respondents stated how many times they had been married, but data on age at end of first marriage were not collected so a full risk analysis was not possible. A similar analysis was carried out to examine the odds of being in a polygamous union as opposed to a monogamous union.

These results were restricted to respondents aged 30–49 years at their last interview. Younger respondents were omitted because respondents who experienced a later AFS and/or spent a longer time sexually active but unmarried would start their first marriages at older ages and thus have less time in which to experience marital break-up or have their first marriage become polygamous. Since almost all respondents (95% of men and 99% of women) were married before the age of 30 years, this restriction ensured that almost all ever married respondents in the sample had had some exposure to risk of changing their marital status to a less favourable one from the point of view of multiple partnerships.

Continuous forms were used for AFS and time spent between sexual debut and first marriage. Categorical (binary) definitions of early AFS and a long period single were explored but would have entailed different definitions for men and women and, in fact, the continuous forms were more stable in stepwise procedures. The assumption of linearity was checked for all combinations of predictor and outcome variables using non-parametric tests for trend, breaking down predictors by discrete single years in all categories with more than 50 subjects each.

We also investigated whether AFS and length of premarital sexual activity influenced the number of sexual partners reported at a later time. In this case, we reported the crude regression analyses and then adjusted for reporting age (measured in single years) and for each marital status category, since we previously determined that the number of sexual partners reported in the last year varied considerably with marital status. Robust standard errors were reported to allow for respondents reporting in more than one survey.

We considered using the integer number of partners reported as the outcome variable and using linear regression; however, the distributions of partners reported were heavily skewed so assumptions of normality would not be satisfied. We therefore created a binary variable to define a higher risk group for number of partners reported and used logistic regression. Different cut-off points were chosen for men and women to define high-risk groups. Women who reported two or more partners in the last year (10% of the sexually active population) were considered to be in a high-risk group, while for men the high-risk group was defined as having three or more partners in the last year (28% of the sexually active population). The results were calculated for all respondents aged 15–49 years; younger respondents were not excluded from this analysis as the dependant variable refers to events in the last 12 months as opposed to being an “achieved status” variable for which risk accumulates over time. To investigate the effect of interactions between marital status, age and the two predictor variables, we repeated the analysis for each marital status group separately.

The statistical package Stata Version 9 (StataCorp, College Station, Texas, USA) was used in the analysis.

## RESULTS

### Consistency of age reporting

The total number participating in at least one of the four surveys and providing information about marital status or sexual activity was 16 832, of whom 15 415 had ever had sex and 11 956 were ever married by the last round. Of those interviewed in the first round, 73% were followed up and interviewed again in a later survey, 26% moved out or died before round 4, and 1% refused to participate in any of the later survey rounds.

Current status information (available in all four rounds) was very consistent, with only 101 individuals (71 men and 30 women) reporting that they had never had sex after previously reporting they had, and 106 individuals (43 men and 63 women) reporting they were never married having previously reported being married. The consistency of current status reports is thus over 99%.

With AFS, age consistency checks were possible for 4028 individuals (1761 men and 2267 women) who participated in round 4 and at least one other earlier survey and had become sexually active by round 3. Similar checks for consistency of reporting AFM were possible for 3550 individuals (1386 men and 2164 women) who had married by the time of their round 4 interview. Altogether, 37% of men and 28% of women gave inconsistent reports of AFS, and 36% of men and 31% of women gave inconsistent information about AFM.

Quality of reporting of both AFS and AFM declined with age for both sexes: 83% of women and 77% of men born after 1980 gave consistent reports of AFS in rounds 1 and 4, and 76% and 79% of these youngest men and women reported consistently about AFM. By comparison, in the cohort born 1950–9, only 44% of men and 65% of women reported AFS consistently, and 50% of men and 61% of women gave consistent reports on AFM.

**Figure 1:**
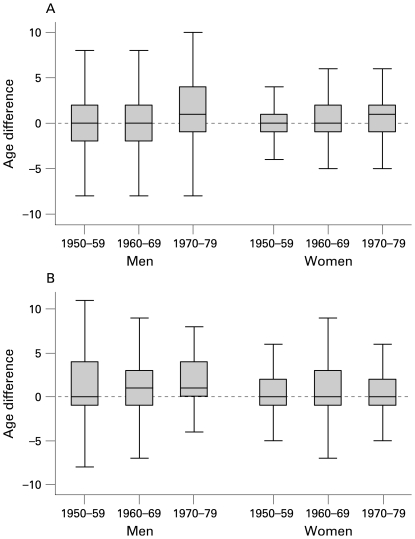
(A) Differences in reported age at first sex (AFS) (round 4 − round 1) and (B) differences in reported age at first marriage (AMS) (round 4 − round 1).

For individuals reporting AFS or AFM in both surveys, we calculated the difference (round 4 report – round 1 report) and examined the distribution of these differences by sex and 10-year birth cohort; 37% of men and 51% of women reported consistently (within ±1 year) their AFS, with 24% of men and 21% of women reporting AFS >1 year higher in round 1 than in round 4, while 40% of men and 28% of women reported an AFS >1 year higher in round 4 than in round 1. AFM was consistently reported by 43% of men and 46% of women, with 21% of either sex reporting a significantly lower AFM in round 4, whereas 36% and 33% of men and women, respectively, reported significantly higher AFM in round 4. A visual inspection of the data (fig 1A and B) suggests that the aggregate reporting bias due to inconsistent age reporting must be very small, since the median age differences are all close to zero. There is a slight upward skew in some of the male distributions, suggesting that men born in later birth cohorts have a tendency to report older ages at the second interview than those in earlier birth cohorts. The spread of age differences is wider for men than for women.

The nature of the inconsistencies suggested random errors rather than persistent bias. AFS and AFM reported at the later round were almost as likely to be lower as higher. Correction of inconsistent age reporting by averaging the inconsistent reports produced life table curves for AFS and AFM which were virtually identical to those based only on consistently reported ages. Rather than omitting respondents with inconsistent age reporting, the inconsistent ages were corrected by averaging and these corrected values were used in the following analysis.

### Trends in AFS and AFM

**Figure 2:**
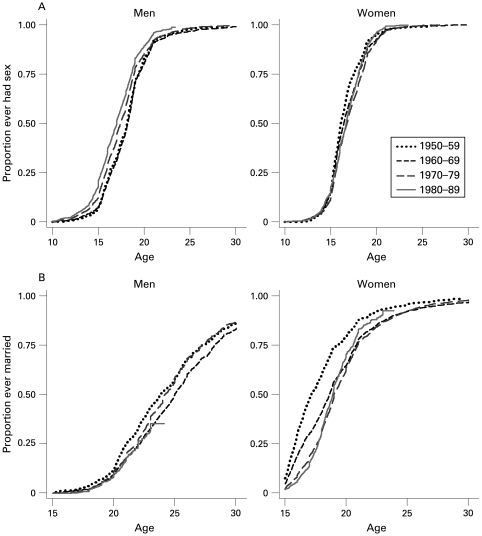
Pattern of (A) age at first sex (AFS) and (B) age at first marriage (AFM) by sex and birth cohort.

Four 10-year birth cohorts are compared in figs 2A (AFS) and 2B (AFM) from those born in 1950–9 to those born in 1980–9. AFS has become progressively earlier for men (median declined from 18.1 years for the oldest cohort to 17.0 years for the youngest), but slightly later for women (median increased from 16.2 years in the oldest cohort to 16.8 years for those born in 1970–9, falling slightly to 16.6 years for women born in 1980–9). AFM has also become markedly later for women (median increased from 17.2 years to 18.9 years) whereas, for men, AFM has vacillated (median age was 23.8 years for those born in 1950–9, 24.9 years for those born in 1960–9 and 24.1 years for those born in 1970–9). Median AFM cannot yet be ascertained for the youngest male cohort. The time between sexual debut and marriage has become progressively longer in younger cohorts for men and women.

**Figure 3:**
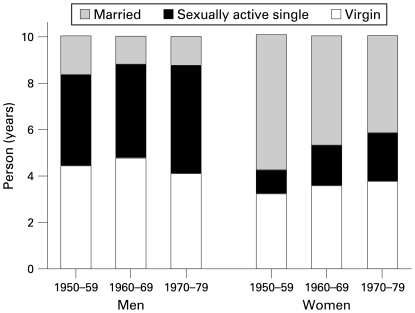
Person-years between ages 15 and 25 years for each sexual activity status by sex and birth cohort.

Focusing only on persons aged ⩾25 years at their last interview, fig 3 shows the distribution of person-years between 15 and 25 years by sexual activity status and birth cohort for men and women. The time spent between sexual debut and first marriage is much longer for men (overall mean across all cohorts 3.6 years) than for women (mean 1.6 years). Time spent sexually active but not yet married increased steadily from earlier to later cohorts, from 3.9 to 4.7 years for men and from 1.0 to 2.1 years for women for cohorts born in 1950–9 and 1970–9, respectively.

**Figure 4:**
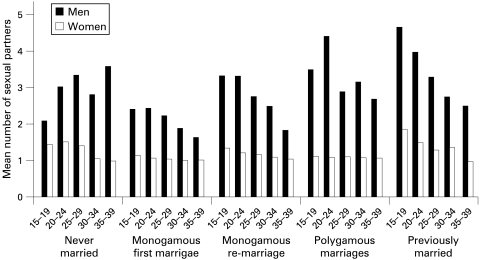
Mean number of partners in last year (four rounds combined excluding virgins) by sex, age group and marital status.

Figure 4 (which excludes those who have never had sex) shows that, for every age group and marital status category, men reported more partners in the last year than women: the overall mean for men across all age groups and marital status categories was 2.2 partners in the last year compared with 1.1 for women. Monogamous marriages are shown subdivided into first and subsequent unions, but polygamous unions are not subdivided. For clarity, this graph has been truncated at 40 years to avoid erratic fluctuations due to small numbers in the older age groups. For both sexes and in all marital status categories, the number of partners in the last year decreased with age except for single men who reported more partners at older ages. There was no significant change in any of these patterns over time (between rounds), and data for all four rounds are shown combined. This means that some respondents contribute more than once, at different ages, and possibly in different marital status categories.

Men and women in monogamous first marriages had the lowest mean number of partners: 2.0 for men and 1.0 for women after standardising for age. Among men, the previously married (currently widowed, divorced or separated) and those in polygamous marriages had the highest numbers of partners (mean 3.2 after standardising for age); remarried and single men had an age-standardised average of 2.6 partners. For women, the highest mean numbers of partners were reported by previously married and single women (1.3), followed by those who had remarried or entered polygamous unions (1.1).

### Relationship between sexual behaviour in youth and in later life

Table 1 shows the relationship between AFS and time spent sexually active but not yet married (measured in years) with marital instability in later life: separation or widowhood (either of which may be followed by remarriage) and polygamous marriage. Non-parametric tests for trend on single year values all yielded highly significant z-scores, with (pr |z| <0.025) for both sexes, and all combinations of predictor and outcome variables.

**Table 1:** Association between age at first sex (AFS) and time from sexual debut to marriage with subsequent marital breakdown and polygamy

Outcome and population	Predictor variable	Unadjusted estimates	Estimates adjusted for age last birthday and other predictor
		Proportionate effect on OR of 1 year increase in predictor (95% CI)	Proportionate effect on OR of 1 year increase in predictor (95% CI)
Marital instability			
Men			
N = 3385	AFS	0.98 (0.95 to 1.01)	0.97 (0.94 to 0.99)
N = 3292	TSSSA	0.98 (0.96 to 0.99)	0.97 (0.95 to 0.99)
Women			
N = 5064	AFS	0.91 (0.89 to 0.94)	0.91 (0.89 to 0.94)
N = 4991	TSSSA	0.98 (0.96 to 1.00)	0.98 (0.96 to 1.00)
Polygamy			
Men			
N = 3084	AFS	0.94 (0.90 to 0.99)	0.88 (0.84 to 0.93)
N = 3011	TSSSA	0.92 (0.89 to 0.96)	0.90 (0.87 to 0.93)
Women			
N = 4220	AFS	0.96 (0.92 to 0.99)	0.95 (0.91 to 0.98)
N = 4165	TSSSA	0.95 (0.92 to 0.98)	0.95 (0.93 to 0.98)

AFS, age at first sex; CI, confidence interval; OR, odds ratio; TSSSA, time spent single and sexually active.

The results suggest that men and women who have a later AFS and those who spend longer in the “sexually active unmarried” state are less likely to experience marital breakdown. For men, after adjusting for age, an increase of 1 year in either AFS or time single gives a 3% reduction in the odds of experiencing marital breakdown after the age of 30 years. For women, an increase of 1 year in AFS is associated with a 9% reduction in the odds of experiencing marital breakdown, whereas a year spent sexually active and single affords a 2% reduction in these odds.

For polygamy the association is even stronger: men who experience later AFS and stay single longer are less likely to be in polygamous marriages after the age of 30 years; the results are highly significant and the two factors reinforce each other, with a 12% reduction in the odds of polygamy for a 1-year delay in AFS and a 10% reduction for a year less spent single and sexually active after adjusting for age. For women, each year’s delay is associated with a 5% reduction in the odds of entering a polygamous marriage as first or second wife. Adjusting for age and for the other predictor variable marginally improves the significance and magnifies the impact of each predictor variable.

Table 2 shows the relationship between AFS and length of time spent sexually active and unmarried and the number of partners reported at a later time. The results are very consistent and similar for both sexes: later AFS is protective whereas a longer time spent sexually active and unmarried increases the risk of having a larger number of partners later in life. The crude and adjusted odds ratios (OR) are broadly similar, and there is little loss of significance after adjusting for reporting age, marital status and the other predictor variable.

**Table 2:** Association between age at first sex (AFS) and time from sexual debut to marriage with subsequent number of partners reported in last 12 months

Population and outcome	Predictor variable	Unadjusted estimates	Estimates adjusted for age last birthday, marital status and other predictor
		Proportionate effect on OR of 1 year increase in predictor (95% CI)	Proportionate effect on OR of 1 year increase in predictor (95% CI)
Men reporting ⩾3 partners			
N = 8631	AFS	0.93 (0.91 to 0.95)	0.95 (0.92 to 0.97)
N = 8408	TSSSA	1.04 (1.02 to 1.05)	1.03 (1.01 to 1.05)
Women reporting ⩾2 partners			
N = 11106	AFS	0.90 (0.86 to 0.93)	0.93 (0.89 to 0.96)
N = 10754	TSSSA	1.10 (1.08 to 1.12)	1.07 (1.04 to 1.09)

AFS, age at first sex; CI, confidence interval; OR, odds ratio; TSSSA, time spent single and sexually active.

The adjusted ORs show that, for men, each year’s delay in sexual debut lowers the odds of subsequently reporting more than two partners in the last year by 5%, whereas each additional year spent single and sexually active raises the odds by 3%. For women, the effects are slightly larger: each year’s delay in sexual debut lowers the odds of having more than one partner in the last year by 8%, whereas each year spent single and sexually active raises these odds by 7%.

Repeating this analysis separately for each marital status group to investigate interactions, we found that, for men, the adjusted ORs for AFS varied between 0.91 (95% CI 0.87 to 0.94) in the never married group and 0.98 (95% CI 0.92 to 1.04) in the monogamously remarried group. For time single and sexually active, the OR varied between 1.02 (95% CI 0.96 to 1.08) for previously married men and 1.06 (95% CI 1.03 to 1.08) for those who had never married, with the loss of significance mainly due to small numbers in some categories. For women, the adjusted OR for AFS varied between 0.90 (95% CI 0.83 to 0.96) for single women to 0.95 (95% CI 0.85 to 1.07) for those polygamously married. The OR for time spent single and sexually active ranged from 1.01 (95% CI 0.96 to 1.06) among never married women to 1.12 (95% CI 1.08 to 1.18) for those in monogamous first marriages.

In the case of those who were not yet married, current age (rather than age at marriage) defined the upper limit of time spent single, so AFS, time spent single but sexually active and current age cannot be used in a single regression; the ORs for the never married subjects quoted above are therefore only adjusted for reporting age, not for the other predictor variable.

## DISCUSSION

This analysis has shown that, although we can detect reporting inconsistencies in retrospective reports of AFS and AFM, it is possible to make simple corrections (averaging pairs of inconsistent reports) which do not appear to bias the data at an aggregate level. Almost all discrepancies were caused by respondents reporting slightly different ages at different interview times; there were very few instances of inconsistent reports of current status (eg, claiming never to have married after reporting being married at an earlier round). There is little indication of systematic bias in retrospective reporting over time: inconsistencies go in either direction, correction through averaging may introduce random noise (if one of the reported ages was in fact correct) but is unlikely to introduce bias into estimates of trend in timing of sexual debut and marriage.

Some of the age inconsistencies may stem from enumerators trying to help respondents figure out their current age at the interview date when only their year of birth (not the day or month) are known. Such problems will be less important in more recent cohorts because a larger proportion of those born in the 1980s and 1990s know their exact dates of birth.

We found that, on average, men spend more than twice as long as women between their first sexual experience and their entry into a first marital union. As the “single but sexually active” state is characterised by a fairly high rate of partner acquisition,^23^^–^28 men would inevitably acquire more lifetime partners by the time they married than women, even if their propensity to form new partnerships in this interval was similar to that of women. In fact, the average number of sexual partners in the last 12 months reported by single sexually active men was about twice as high as the number reported by women, so that the higher rate of partner acquisition by men exacerbated the effect of the longer duration that they spend single and sexually active.

**Take-home messages**

- Inconsistent reporting of age at first sex (AFS) and age at first marriage (AFM) is widespread, but introduces noise rather than age-related bias.
- AFS for men has fallen and AFM for women has risen, so both sexes spend longer sexually active but unmarried.
- Early age at marriage is statistically associated with marital instability.
- Longer time spent sexually active and unmarried is statistically associated with higher rates of partner acquisition at later stages of life.

The time interval “single but sexually active” is increasing in length for both men and women because AFS is falling for men and AFM is rising rapidly for women. This lengthening trend carries with it the danger of increasing numbers of men and women entering first marriage already infected with HIV, as they will have had more time to acquire new sexual partners before marriage, even if the rate of acquisition of partners by sexually active singles did not change over time.

As well as accelerating an individual’s exposure to the “single but sexually active” state, early sexual debut is associated with a higher propensity to be found, in later life, in marital status categories in which there is a higher rate of multiple partner acquisition (polygamous marriages, remarriages, and being separated, widowed or divorced). Independently, shorter time in the “single but sexually active” state is also associated with a higher likelihood of experiencing marital breakdown later in life. This may suggest that marriages contracted at young ages are more prone to fall apart, as has previously been found in developed countries.^43^ However, we cannot infer this directly from our data as we do not yet have estimates for the dates of ends of marriages; ideally, a survival analysis for first marriages is required to ascertain this link.

The analysis of reports of sexual partners in the year preceding each survey round also revealed that early sexual debut and longer time spent sexually active but single were independently associated with a propensity to form multiple sexual partnerships for both men and women. This effect is independent of the propensity to enter high-risk marital status categories since it persists when marital status is controlled in multiple regression or when separate regressions are done for each marital status category. The same findings hold for men and women.

Our analyses raise interesting questions that should be investigated further in longitudinal studies, possibly with qualitative components. For example, the observation that previously married men and women (separated and widowed) have relatively large numbers of partners raises the question whether they tend to have large numbers of partners because their current status offers them the opportunity (or, in the case of women, denies them the economic support provided by a spouse), or whether it was their propensity to form multiple partnerships that led to the breakdown of their previous marriage. A more general question also arises as to whether there is evidence for the existence of a “sexually adventurous” personality type who is always drawn to acquisition of new partners. We cannot use the Kisesa data in their current form to examine this as we do not know whether the partners reported in the last 12 months are of long standing or newly acquired. Data on the start and end dates of relationships are needed for this, and such data would also allow us to estimate the extent of concurrent relationships in this population.

The existence of “personality types” that are characterised throughout life by an appetite for sexual adventure could be explained by biological factors (hormone balance) or early childhood socialisation in the family (parental example). However, it has also been suggested that the explanation for the strong association between premarital sexual activity and extramarital partnerships in later life is the act of experiential learning about sexuality that occurs during adolescence which has profound lifelong effects.^1^ ^34^ ^44^ If this is the case, then interventions that succeed in influencing premarital behaviour in young people could have a much more important and long-lasting effect on their subsequent behaviour, and hence play a really important part in HIV prevention.
